# Motor Conduction Studies and Handgrip in Hereditary TTR Amyloidosis: Simple Tools to Evaluate the Upper Limbs

**DOI:** 10.3389/fneur.2022.835812

**Published:** 2022-02-28

**Authors:** Vincenzo Di Stefano, Ewan Thomas, Valerio Giustino, Salvatore Iacono, Angelo Torrente, Guglielmo Pillitteri, Andrea Gagliardo, Antonino Lupica, Antonio Palma, Giuseppe Battaglia, Filippo Brighina

**Affiliations:** ^1^Department of Biomedicine, Neuroscience and Advanced Diagnostic (BIND), University of Palermo, Palermo, Italy; ^2^Department of Psychology, Educational Science and Human Movement, University of Palermo, Palermo, Italy

**Keywords:** TTR, hereditary amyloid neuropathy, median nerve, handgrip, hand strength, carpal tunnel syndrome, neurophysiology, nerve conduction study - NCS

## Abstract

**Purpose:**

Hereditary transthyretin amyloidosis with polyneuropathy (ATTRv) is caused by mutations in the *TTR* gene, leading to misfolded monomers that aggregate generating amyloid fibrils. The clinical phenotype is heterogeneous, and characterized by a multisystemic disease affecting the sensorimotor and autonomic functions along with other organs.

**Materials and Methods:**

All the patients were assessed by complete neurological assessment, neurophysiological evaluation, of the median nerve, and handgrip analysis. The data are presented as means and standard deviations. Parametric and non-parametric assessments have been performed to identify differences between groups. Pearson's correlation has been carried out when appropriate.

**Results:**

Twenty patients with ATTRv (66.1 ± 8.4 years; eight females) and 30 controls (61.1 ± 11.6 years; 16 females) were enrolled. Handgrip strength was reduced in patients with ATTR in both right and left hands compared to the controls. Significant differences were found between patients and controls in the right (handgrip right, HGS_R_ TTR 21.1 ± 13 kg vs. HGS_R_ Control 29.4 ± 11.3 kg, *p* = 0.017) and left (handgrip left, HGS_L_ TTR 22.2 ± 10.7 kg. vs. HGS_L_ Control 31 ± 11.3 kg, *p* = 0.007). NIS and CMAP amplitude of the median nerve were related to HGS measures for both hands in patients with ATTRv.

**Conclusions:**

The progression of bilateral carpal tunnel syndrome is related to neurophysiological data in the median nerve in ATTRv. Also, handgrip measures might represent an important tool for the assessment of disease progression in ATTRv. We propose using a combination of CMAP amplitude and HGS for the assessment of hand motor strength in ATTRv.

## Introduction

Hereditary transthyretin amyloidosis with polyneuropathy (ATTRv) is caused by mutations in the *TTR* gene, leading to misfolded monomers that aggregate generating amyloid fibrils (1). The clinical phenotype is heterogeneous, and is characterized by a multisystemic disease affecting the sensorimotor and autonomic functions along with other organs.

Carpal tunnel syndrome (CTS) is a well-known symptom of ATTRv and appears very early in the disease course, causing hand clumsiness and sensory deficits associated with weakness and hypotrophy in tenar muscles (2). Also, CTS can be the first sign of the disease and is often present in middle-aged carriers of *TTR* variants. Moreover, hand weakness progresses along with aggregation of amyloid fibrils in peripheral nerves and the carpal ligament (2). As a consequence, patients with ATTRv often complain of severe neuropathy in the median nerve with significant limitations and implications in the quality of life due to difficulties in the execution of simple movements, such as turning a key in a lock or signing a document.

It is a well-known fact that ATTRv, if untreated, can lead to death within 7–10 years of disease onset (1); however, recent advancements in diagnosis and treatment of this unfavorable condition have surprisingly improved so much that patients with ATTRv might not only survive longer but also enjoy better prognosis and quality of life (3, 4). Indeed, while the first therapies discovered aimed to stabilize the tetramer of TTR (even if new amyloid fibrils might accumulate in tissues of patients), the most recent drugs for the treatment of ATTRv are based on RNA-interference mechanisms, and they almost completely stop the production of TTR as a result of complete stabilization of the disease and mild recovery from symptoms of neuropathy (4–6). These unprecedent successes on the treatment of ATTR demand more accurate and punctual evaluation of ATTRv, even with the aid of specific tools. Conventional neurophysiology is routinely performed on patients with ATTRv patients showing length-dependent axonal sensorimotor polyneuropathy that usually starts in the lower limbs and subsequently extends to the upper limbs (7). However, the involvement of the upper limb is early and begins in the median nerve with CTS even decades before disease onset (8).

Handgrip strength (HGS) measurement is an instrument that allows for the estimation of overall muscle strength, a physical characteristic strictly connected to carrying out of activities of daily living (ADL) (9). Indeed, decrease in handgrip strength, common in some conditions such as carpal tunnel patients, can negatively affect the quality of life (QoL) (10). Moreover, it is widely recognized that HGS represents a valid marker for physical health, and it is considered a predictor for risk of falls in the elderly (11).

In this study we explored the role of nerve conduction studies (NCSs) and HGS for the assessment of strength in patients with ATTRv, and compared our results with those of healthy controls.

## Methods

### Aims of the Study

In this study, we described the electrophysiological features of the median nerve and strength assessed by handgrip evaluation in a cohort of patients with ATTRv to evaluate the combined use of neurophysiology and handgrip measures to assess the strength of the upper limbs.

### Clinical Evaluation

The severity of ATTRv polyneuropathy was assessed with the Neuropathy Impairment Score (NIS), a scale assessing three major components (muscle weakness, muscle stretch reflexes, and sensation) in the head and upper and lower limbs (7). We calculated the total score (NIS, muscle weakness, muscle stretch reflexes, and sensation), the motor component alone for the whole body (NIS-W, muscle weakness), and the composite subscore by anatomic region for the upper limbs (right and left NIS-W_UL_).

### Electrophysiology Procedures

Nerve conduction studies (NCSs) were performed on both median nerves for all the subjects enrolled according to standard procedures (i.e., bipolar surface stimulating electrodes delivering rectangular pulses 0.1–0.5 ms in duration and recording electrodes placed over the recording site with a ground electrode placed between recording and stimulation electrodes) (12). The stimulation of the right and left median nerve was performed at wrist and elbow and recording from APB. Distal motor latency (DML), compound motor action potential (CMAP) amplitude and motor conduction velocity (MCV) were analyzed in the NCS.

### Handgrip Samples and Data Analysis

**Figure 1:**
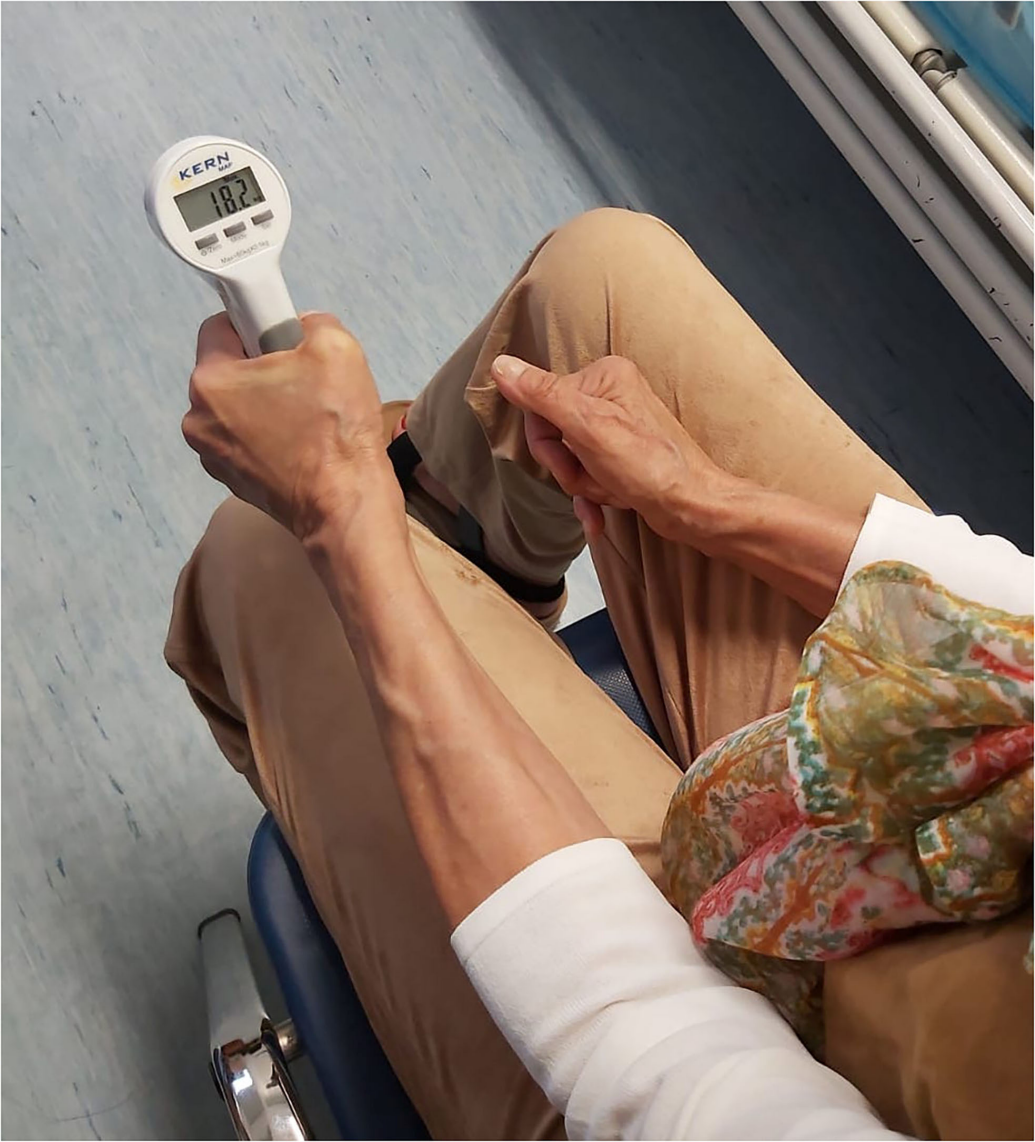
A subject during a handgrip test of the left hand.

Maximal isometric handgrip strength for both right and left hands was measured using a mechanical dynamometer in units of kgf (KernMap model 80K1 - Kern^®^, Kern & Sohn GmbH, Balingen, Germany) in the standardized position recommended by the American Society of Hand Therapists (13), that is, sitting in a chair with a 90° backrest and 90° elbow flexion of the hand to be evaluated (Figure 1). Each participant exerted three trials of 3 s of maximal isometric handgrip strength alternatively for the right and left hands, with a rest period of 180 s between trials and considering the best performance of the three trials for statistical analysis.

### Statistics

Data are presented as means and standard deviations. Shapiro-Wilks was performed to test for normality. Parametric (unpaired Student's *t*-test) and non-parametric assessments (Mann Whitney *U* rank tests) were performed to identify differences between groups. Furthermore, Pearson's correlation was carried out on the TTR amyloidosis group to identify associations with clinical variables. Significance was set to *p* < 0.05 for all analyses. All the analysis were carried out with Jamovi (The jamovi project 2021; jamovi version 1.8.0.1, computer software, retrieved from https://www.jamovi.org). Graphs were created with GraphPad Prism8 (GraphPad Software, San Diego, CA, United States).

## Results

### Patient Demographics and Clinical Features

Twenty patients affected by TTR amyloidosis (66.1 ± 8.37 years, eight females) and 30 controls (61.1 ± 11.56 years, 16 females) participated in the study. Genetic testing confirmed a mutation in heterozygosis in the *TTR* gene in all the patients enrolled. F64L (p.F84L) mutation was encountered in 15 patients, followed by E89Q (p.E109Q) in two patients, V122I (p.V142I) in two patients, and H90A (p.H110A) in one patient. The most frequent symptoms were carpal tunnel syndrome (50%), cardiomyopathy (44%), weight loss (38%), and autonomic dysfunction (28%). Controls have been selected among patients' relatives (i.e., brothers, sisters, sons and daughthers) who screened negative to genetic testing for *TTR* mutations. All the patients underwent complete neurological assessment, neurophysiological evaluation, and handgrip analysis. No significant differences were retrieved for anthropometric characteristics between the two groups (Table 1).

**Table 1:** Descriptive characteristics of the participants.

	**Age (years)**	**Height (cm)**	**Weight (kg)**
TTR	66.1 ± 8.37	166.2 ± 10.90	70.5 ± 18.56
Controls	61.1 ± 11.56	166.1 ± 9.02	77.2 ± 12.07

*Data are presented as means ± standard deviation*.

### Clinical Evaluation

Fifteen patients presented mild symptoms (FAP I), and five had moderate to severe symptoms (FAP II). Patients with ATTRv presented a mean NIS of 37.15 ± 30.71 and NIS-W of 18.54 ± 22.4. In the right upper limb, NIS-W_UL_ scored 3.83 ± 5.49, while in the left it was 3.61 ± 4.9.

### Handgrip Measures

**Figure 2:**
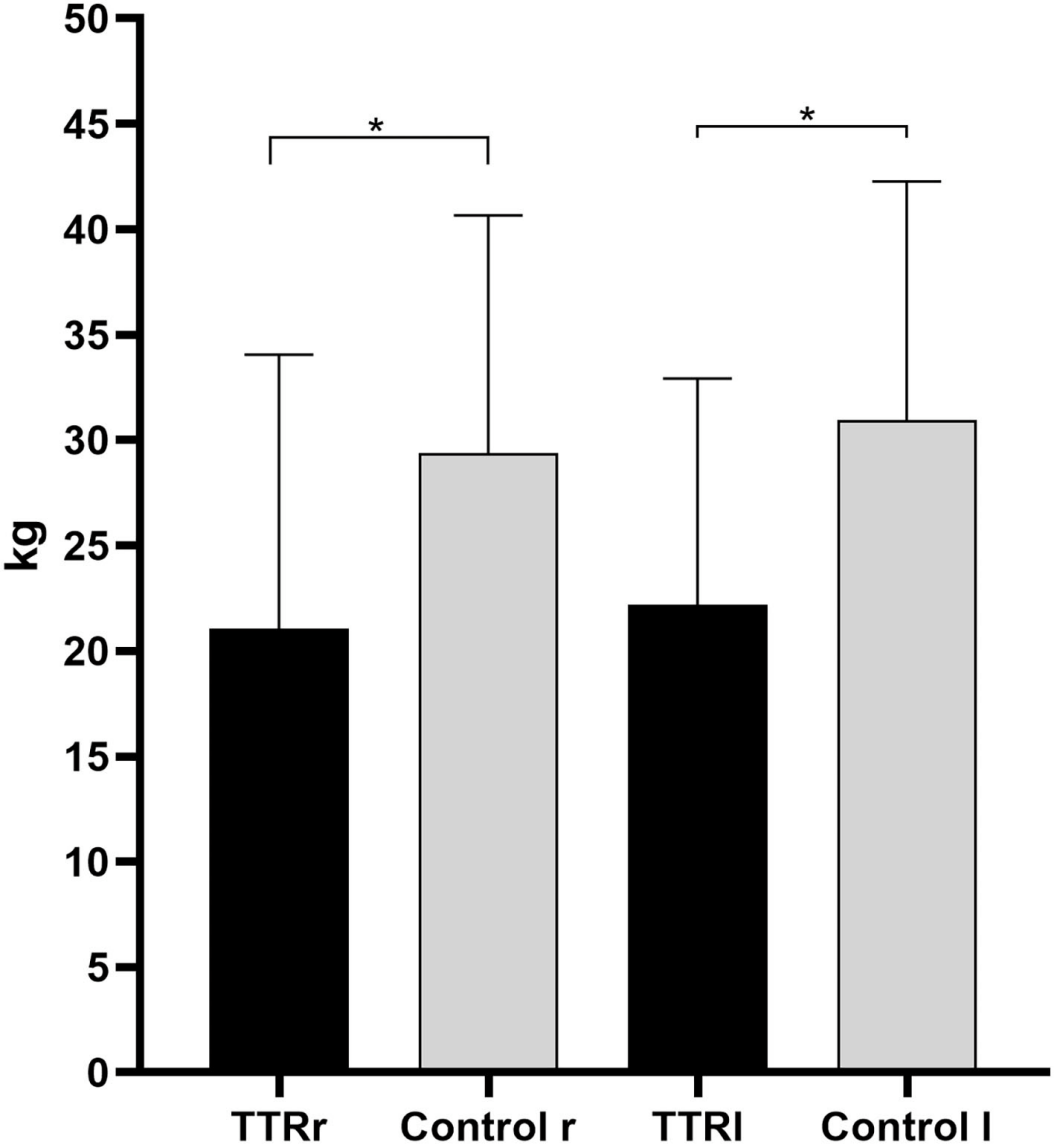
Handgrip measurements of patients with hereditary transthyretin amyloidosis with polyneuropathy (ATTRv) and controls for the right and left hands. TTRr, handgrip strenght in the right; TTRl, handgrip strenght in the left; r, right; l, left. ^*^Mann-Whitney U-test, *p* < 0.05.

When the grip strength of both hands was evaluated, significant differences in the right (handgrip right, HGS_R_, TTR 21.1 ± 13 kg vs. Control 29.4 ± 11.3 kg, *p* = 0.017) and left (handgrip left, HGS_L_, TTR 22.2 ± 10.7 kg vs. Control 31 ± 11.3 kg, *p* = 0.007, Figure 2) were retrieved between the patients and the controls.

### Neurophysiological Data

In the right median nerve, a significant difference was reported between the patients with ATTRv and controls for DML and MCV (Table 2). In the left median nerve, the difference was more pronounced and demonstrated in all the parameters (DML, CMAP amplitude, and MCV). A significant correlation was found between the right median nerve DML and CMAP amplitude (−0.34, *p* = 0.049), as expected, because of the presence of CTS.

**Table 2:** Neurophysiological variables relative to the EMG of the median nerve in patients with hereditary transthyretin amyloidosis with polyneuropathy (ATTRv) and controls.

	**ATTRv patients**	**Controls**	***P*-value**
**Right median nerve**			
DML (ms)	4.4 ± 1.6	3.0 ± 0.4	<0.0001^*^
CMAP Amplitude (mV)	7.4 ± 5.0	8.9 ± 2.2	0.66
MCV (m/s)	48.4 ± 5.0	56.6 ± 5.1	<0.0001^*^
**Left median nerve**			
DML (ms)	4.4 ± 1.6	3.2 ± 0.7	0.012^*^
CMAP Amplitude (mV)	5.6 ± 2.9	8.9 ± 1.0	0.001^*^
MCV (m/s)	48.2 ± 4.4	53.4 ± 2.5	<0.0001^*^

*Data are presented as means ± standard deviation*.

*NA, not applicable; FAP, familial amyloid polyneuropathy; TTR, transitiretine; DML, distal motor latency; CMAP, compound motor action potential; MCV, motor conduction velocity*.

* *Mann-Whitney U-test, p < 0.05*.

### Correlations Among Clinical, Neurophysiological, and Handgrip Measures

Clinical and neurophysiological variables for the ATTRv group (described in Table 2) have been further analyzed and related to HGS.

### Correlations Between Clinical and HGS Measures

Total NIS score was negatively correlated with both HGS_R_ (*r* = −0.45, *p* = 0.033) and HGS_L_ (*r* = −0.5, *p* = 0.015). Also, the motor subscore NIS-W was negatively correlated with HGS_L_ (*r* = −0.44, *p* = 0.035); a similar correlation was present for the right side, but it was not statistically significant (*r* = −0.39, *p* = 0.06). NIS-W_UL_ scores were negatively correlated with homolateral HGS measured for both the right (*r* = 0.43, *p* = 0.039) and left upper limbs (*r* = −0.46, *p* = 0.027).

### Correlations Between Clinical and Neurophysiological Measures

Total NIS score was negatively correlated with CMAP amplitude of both right (*r* = −0.58, *p* = 0.014) and left (*r* = −0.61, *p* = 0.01) median nerves. The motor subscore NIS-W was negatively correlated with the CMAP amplitude of the left median nerve (*r* = −0.44, *p* = 0.035). NIS-W_UL_ scores were not correlated with any neurophysiological data.

### Correlations Between Neurophysiological and HGS Measures

The CMAP amplitude of the right median nerve showed a positive correlation with HGS_R_ (*r* = 0.54, *p* = 0.026). Similarly, we found a positive correlation between the CMAP amplitude of the left median nerve and HGS_L_ (*r* = 0.56, *p* = 0.02). No significant correlations were established between DML and VCM of both median nerves and HGS_R_ or HGS_L_.

## Discussion

Nowadays, hereditary transtiretin amyloidosis is a tractable disorder, for which a prompt diagnosis is needed, and biomarkers are on demand (14). Many molecules have been studied for cardiac phenotypes, such as serum retinol-binding protein 4 or B-type natriuretic peptide and transtiretin (15). However, there are only a few studies on biomarkers for nerve damage in ATTRv. Of interest, serum neurofilament light chain has been recently studied in ATTRv (16); however, there are only preliminary results available (14), that show a correlation with the severity of polyneuropathy, thus proposing NfL as a biomarker for nerve damage.

Handgrip test is a rapid, simple, and non-invasive tool that can be used for strength evaluation in patients affected by neuromuscular diseases. Furthermore, it is an attractive tool, because it can be easily performed and does not require expensive equipment (Figure 1). Several studies have shown how handgrip test is altered in polyneuropathies, such as diabetic one, in which patients show a significant reduction of strength (17). Moreover, some authors tried to use it as an outcome measure, showing promising results in CMT (18). However, to date, few authors have performed handgrip test in the evaluation of familial ATTRv showing significant strength reduction (19).

Here, we aim to confirm alteration in handgrip strength in patients affected by ATTRv and correlate HGS data with clinical and neurophysiological findings. In particular, given the high prevalence of carpal tunnel syndrome in ATTRv (2), we argued to correlate the reduced strength due to CTS with neurophysiological alterations NCS of the median nerve.

The present study directly compares neurophysiological data recorded from the median nerve and HGS in patients affected by ATTRv and healthy controls. HGS demonstrated a significant reduction of strength in both the right and left hands in patients compared to controls (Figure 1); also, the entity of strength reduction was similar in the right and left sides. Moreover, The NCSs showed prolonged DML in both right and left median nerves in the patients but not in the controls (Table 2), while CMAP amplitude was different only in left median nerves of the patients ATTRv (*p* = 0.012). This result might be explained by the high prevalence of CTS in the dominant hand (more often right) of the general population, because the controls also presented a similar mean right CMAP amplitude of the median nerve. Moreover, when present, CTS in ATTRv is usually bilateral, underlining the importance of detecting left (non-dominant) or bilateral CTS in ATTRv as a specific finding of amyloidosis, less often encountered in routine NCS (2, 12). Indeed, while bilateral CTS is an early sign of ATTRv, CTS in the dominant hand is the rule in general population, especially in the elderly (20).

Our data showed that the combined use of neurophysiology and HGS might represent a simple and unexpensive way to assess for motor compromise in ATTRv. As expected, the DML of the right median nerve was negatively correlated to CMAP amplitude but not with HGS in patients with ATTRv. Conversely, the CMAP amplitude of both right and left median nerves was positively correlated with left HGS. Indeed, CMAP amplitude is an expression of the number of motor fibers and, consequently, motor units that can generate maximum strength (21). On the contrary, DML depends on direct compression and local demyelination in the median nerve at wrist in CTS, which is not related with ATTRv itself, and it is not related with motor strength. Hence, we can assume that the reduced strength measured by HGS reflects CMAP amplitude in the median nerve.

Of interest is that relevant correlations have been reported between the CMAP amplitude of the median nerve and both NIS and HGS measures. This finding suggests that reduction in CMAP amplitude of the median nerve might cause a consequent increase in NIS scores and reduction of strength measured by HGS. However, when assessing the upper limbs alone (without considering the score from the lower limbs), correlations between clinical and neurophysiological data disappear; on the contrary, the relationship between NIS-W_UL_ and HGS for both the right and left sides persist. These data support the use of HGS measures, suggesting that they might be more sensitive than neurophysiology alone in the assessment of strength in the upper limbs. Indeed, despite being a very sensitive and reliable tool in the clinical onset, conventional neurophysiology might be less reliable in the follow-up, when motor nerves become not elicitable or too altered to allow an indirect evaluation of residual strength (22, 23). From this perspective, HGS may offer the possibility of qualifying residual strength in the hands and appreciate even little changes that might escape with conventional neurophysiology.

Our study presents some limitations. The selection bias and low number of patients might have led to interpretation errors. Also, the evaluation of isolated median nerves might have carried an underestimation of the contribution of the ulnar nerve in hand weakness in ATTRv. Future studies are needed to validate this promising tool in clinical practice.

## Conclusion

Progression of bilateral carpal tunnel syndrome can be shown with neurophysiological data of the median nerve in ATTRv. Also, handgrip measures might represent a simple and cost-effective tool for the assessment of disease progression in ATTR. We propose using a combination of CMAP amplitude and HGS for rapid and simple assessment of motor strength in ATTRv.

## Data Availability Statement

The data that support the findings of this study are available from the corresponding author upon reasonable request.

## Ethics Statement

The studies involving human participants were reviewed and approved by Palermo1. The patients/participants provided their written informed consent to participate in this study.

## Author Contributions

VD, ET, and VG contributed to the conception and design of the study. VD organized the database and performed data collection. SI, AT, AG, and AL collected the clinical and neurophysiological data. ET, GP, and VG evaluated the handgrip data. VD and ET supervised data collection and analysis. ET performed the statistical analysis. VD, SI, AT, ET, and VG wrote the first draft of the manuscript. AP, GB, and FB revised the manuscript. All authors contributed to manuscript revisions, read, and approved the submitted version of the manuscript.

## Funding

Financial support for article processing fees was provided by Alnylam Pharmaceuticals (Cambridge, MA, USA). The funder was not involved in the study design, collection, analysis, interpretation of data, the writing of this article or the decision to submit it for publication.

## Conflict of Interest

The authors declare that the research was conducted in the absence of any commercial or financial relationships that could be construed as a potential conflict of interest.

## Publisher's Note

All claims expressed in this article are solely those of the authors and do not necessarily represent those of their affiliated organizations, or those of the publisher, the editors and the reviewers. Any product that may be evaluated in this article, or claim that may be made by its manufacturer, is not guaranteed or endorsed by the publisher.
